# Histoplasma Endocarditis of the Native Mitral Valve in a Patient With End-Stage Renal Disease: A Diagnostic Challenge

**DOI:** 10.7759/cureus.82246

**Published:** 2025-04-14

**Authors:** Patrick Berchie, Moatamn Skuk, Samuel Dadzie, Baffour Otchere, Zola Nlandu

**Affiliations:** 1 Augusta University/University of Georgia Graduate Medical Education Program, Piedmont Athens Regional Medical Center, Athens, USA; 2 Department of Infectious Diseases, Piedmont Athens Regional Medical Center, Athens, USA

**Keywords:** blood culture-negative endocarditis, fungal endocarditis treatment, histoplasma endocarditis, infective endocarditis, native mitral valve endocarditis

## Abstract

*Histoplasma* is a rare cause of endocarditis. It usually occurs in immunosuppressed patients, but diagnosis can be challenging given its nonspecific clinical and laboratory findings. This report describes the case of a patient with end-stage renal disease and multiple hospitalizations who was diagnosed with blood culture-negative infective endocarditis and subsequently found to have *Histoplasma* endocarditis of his native mitral valve. Our patient’s nonspecific symptoms posed a diagnostic challenge, which resulted in delayed diagnosis and treatment.

## Introduction

Fungal endocarditis represents a rare subset of infective endocarditis (IE), accounting for 1-2% of IE cases [1]. Among fungal causes, *Histoplasma capsulatum* is an uncommon etiology of blood culture-negative endocarditis (BCNE); literature review indicates that it accounts for 6% of cases [2]. *Histoplasma* is the most common endemic mycosis in the United States [3]. Despite its prevalence in certain regions, clinicians may not initially consider *Histoplasma* as a causative microorganism for IE. Here, we describe the case of a patient with end-stage renal disease (ESRD) and immunosuppression from repeated steroid exposure who developed *Histoplasma* endocarditis involving his native mitral valve. 

## Case presentation

A 65-year-old male patient with a history of ESRD on thrice-weekly hemodialysis, anemia of chronic disease, hypertension, and chronic heart failure with preserved ejection fraction presented with acute dyspnea and hypoxia following outpatient hemodialysis. He was adherent to his dialysis schedule and dialyzed via a right internal jugular tunneled catheter. His previous dialysis access was a brachiocephalic arteriovenous (AV) fistula, which was nonfunctional due to a mid-fistula stenosis unamenable to multiple endovascular therapies. He had been scheduled by Vascular Surgery for the conversion of his AV fistula to a graft. 

Prior to this index admission, he had multiple hospital admissions over a five-month period for similar symptoms attributed to volume overload. During that interval, he also experienced recurrent episodes of polyarthritis involving the small joints of the hands and wrists. These episodes were treated as presumed gout with several short courses of oral prednisone. He denied fever, cough, chest pain, visual disturbance, rash, abdominal pain, diarrhea, oral/genital ulcers, or constitutional symptoms. Notably, he also denied intravenous (IV) drug use, previous dialysis access infection, animal exposure, tick bites, or recent travel. He had worked in building construction prior to his retirement two years ago.

On this index admission, he presented shortly after dialysis with a 1.7 L ultrafiltration volume and was hypoxic with an oxygen saturation of 76% on ambient air. This improved to 92% on 3 L of oxygen via nasal cannula. On examination, he was afebrile (temperature 97.7 °F) with blood pressure of 172/73 mmHg, pulse of 93 beats per minute, and respiratory rate of 20 cycles per minute. Lung examination revealed bilateral basilar crackles. No murmur, rubs, or gallops were appreciated on cardiac auscultation, and there were no peripheral stigmata of infective endocarditis. He had no hepatosplenomegaly. His dialysis catheter site was clean with no clinical evidence of infection. Initial labs showed leukocytosis (WBC 14,000/mm^3^), otherwise unremarkable. CT scans of the chest, abdomen, and pelvis were only remarkable for mild pulmonary edema, mildly enlarged mediastinal and axillary lymph nodes. There was no evidence of a focal infectious process. He was admitted for presumed volume overload and possible dialysis-related hypoxia.

**Figure 1:**
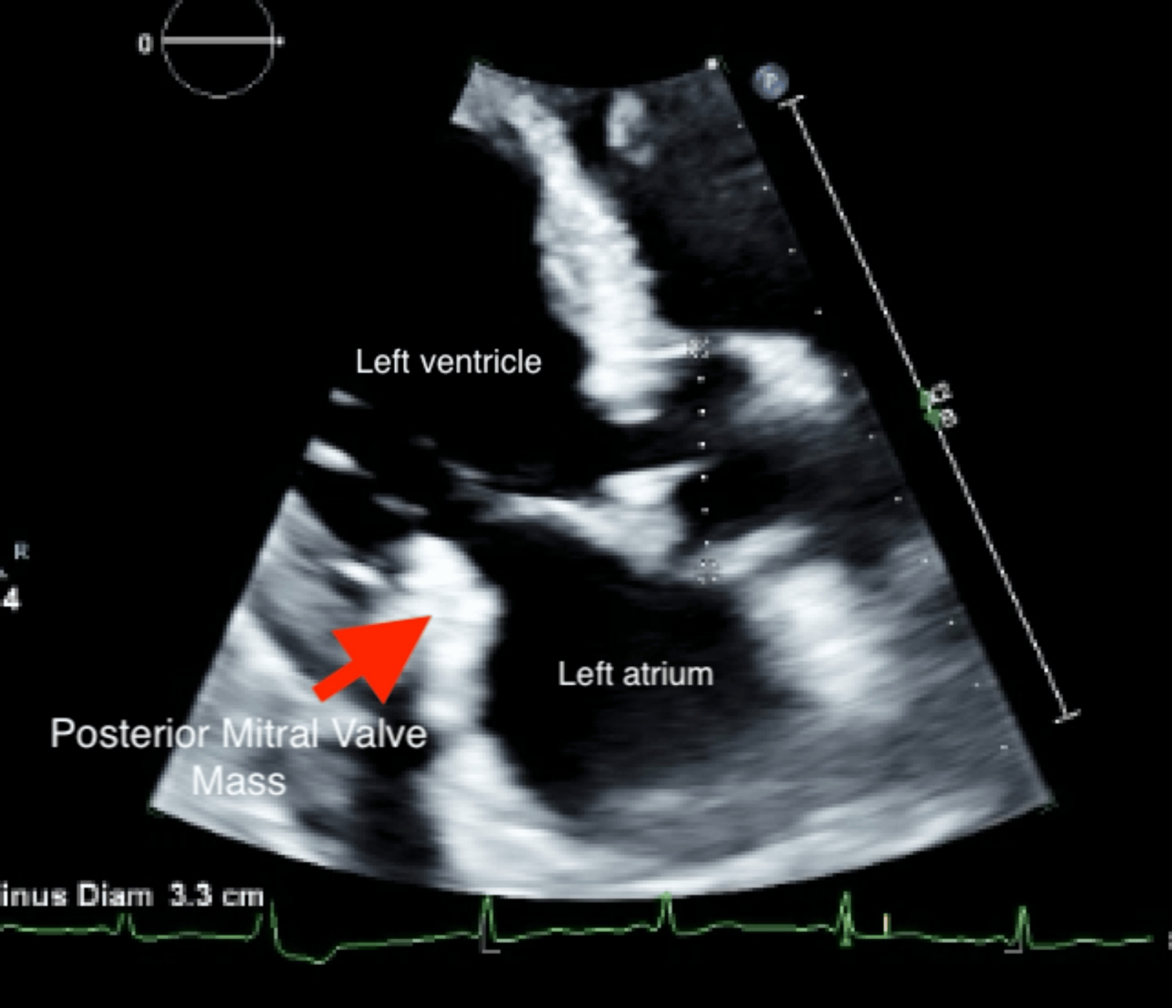
Transthoracic echocardiogram revealing a left ventricular ejection fraction of 50-55% with a large mobile echogenic frond-like irregular multi-lobular mitral valve mass (red arrow) on the posterior valve leaflet with no mitral regurgitation noted.

On hospital day 2, he developed a fever (102 °F) with worsening leukocytosis (peaking at 25,000/mm^3^). Blood cultures were obtained, and empiric IV vancomycin and cefepime were initiated. A transthoracic echocardiogram (TTE) for heart failure evaluation due to initial concern for volume overload showed a markedly enlarged, mobile 2 cm mass on the posterior mitral valve leaflet (Figure 1) without significant regurgitation, raising suspicion for infective endocarditis.

Chart review showed a TTE from five months prior (performed for heart failure assessment), notable for a small, echogenic mass on the posterior mitral valve leaflet, which was thought to be due to calcification. EKG showed normal sinus rhythm with left atrial enlargement and left ventricular hypertrophy. 

Infectious disease was consulted, and a workup for BCNE was initiated. Blood cultures drawn before antibiotic initiation were persistently negative after five days. Serum 1,3-beta-D-glucan, *Aspergillus*, *Histoplasma*, *Coxiella*, and *Bartonella* antibodies were requested. SARS-Cov-2, Influenza A and B, and respiratory syncytial virus (RSV) were negative by polymerase chain reaction (PCR). Serum 1,3-beta-D-glucan was positive. *Histoplasma* antibody by complement fixation was positive (yeast phase titer 1:8, normal <1:8), while *Histoplasma* urine antigen, *Aspergillus *antigen, and serologies for *Bartonella* and *Coxiella* were all negative (Table 1).

**Table 1:** Pertinent laboratory investigations

Laboratory investigation	Results
1,3-Beta-D-glucan	Positive
*Histoplasma* antibodies by complement fixation	Positive 1:8 for yeast phase
*Histoplasma* galactomannan urine antigen	Negative
*Bartonella henselae* antibodies	Both IgM and IgG negative
*Bartonella quintana* antibodies	Both IgM and IgG negative
*Coxiella burnetii* antibodies	Negative
Serum *Aspergillus* antigen by enzyme immunoassay	Negative
HIV Antigen/Antibody test 4^th^ generation	Negative

On day 4, the patient deteriorated, developing hypotension and worsening hypoxic respiratory failure, requiring ICU transfer, intubation, and vasopressor support. Stress-dose corticosteroids were administered for suspected adrenal insufficiency (based on hypotension, hyponatremia of 130 mmol/L, and hyperkalemnia of 5.3 mmol/L). Given his persistently negative blood cultures, lack of response to empiric antibiotics, valvular vegetation, and positive serologic markers, *Histoplasma *endocarditis was deemed likely.

He received one-week induction therapy with IV liposomal amphotericin B, followed by transition to oral itraconazole. He gradually improved over the next 48 hours. While supportive care contributed to hemodynamic recovery, clinical defervescence and stabilization coincided with the initiation of anti-fungal therapy, suggesting a therapeutic response.

Given the large vegetation size and concern for fungal endocarditis, valve surgery was discussed but deferred due to the patient's multi-morbidity and poor surgical candidacy. 

He was discharged in stable condition for outpatient follow-up by cardiology and infectious disease with plans for therapeutic drug monitoring, repeat TTE, and possible long-term anti-fungal suppressive therapy.

## Discussion

Fungal endocarditis is rare, accounting for 1-2% of IE cases [1]. In a review from 1965 to 1995, *Histoplasma* was implicated in 6% of cases [2]. *Histoplasma capsulatum* is the most common endemic mycosis in the United States [3]. It predominantly affects individuals with specific risk factors, including living in endemic areas, occupational exposure, immunosuppression, and presence of prosthetic heart valve [4,5]. Although hyperendemic along the Ohio and Mississippi River Valleys, *Histoplasma* is seen in the entire Eastern half of the United States where our patient resided [6]. It can affect both native and prosthetic heart valves. Diagnosis is usually delayed compared to bacterial endocarditis. In a review of 43 cases, cardiac involvement usually occurred on mitral and/or aortic valves [7]. In a study by Boyanton et al., prosthetic valve endocarditis cases were diagnosed at least 4.1 months earlier than native valve cases [8]. The proposed reason for this delay was heightened surveillance in patients with prosthetic valves.

In the current case, our patient's ESRD, chronic steroid use for presumed gouty arthritis, and likely previous occupational exposure to soil contaminated with bird or bat guano through construction work contributed to his susceptibility. Although he denied recent travel or animal exposure, residing in a region bordering the Ohio River Valley (endemic for *Histoplasma*) increased his risk further. 

Diagnosing *Histoplasma* endocarditis is challenging due to its nonspecific presentation and the low sensitivity of blood cultures with a yield of less than 20% [7]. Serologic testing plays a key role, as demonstrated in this case, where the complement fixation assay provided diagnostic guidance. While the urine antigen test is commonly employed in the diagnosis of histoplasmosis, false negatives can occur; hence, a high index of suspicion must be maintained when clinical parameters suggest a fungal etiology [8]. Although the lung is the portal of entry and site for primary infection, active pulmonary involvement for* Histoplasma* is seldom present at the time of valvular infection [9], as was the case for our patient. In the present case, the discrepancy between the positive serum antibody and negative urine antigen was interpreted as consistent with localized valvular infection or low fungal burden, both scenarios where antigen sensitivity may be reduced. This is a well-documented phenomenon in a multi-center case series where some patients with *Histoplasma *endocarditis had negative urine antigen tests but positive antibody assays [10].

Additionally, this underscores the importance of serologic testing in BCNE. Serologic testing in conjunction with blood culture has been found to yield etiologic identification of BCNE in 8% of cases [11]. Complement fixation has a high positivity rate of 83-92% based on a systematic literature review of 60 cases from 1940 to 2020 [8]. Blood cultures remained persistently negative in our patient. *Histoplasma *antibody may be positive in serum while urine antigen is negative as in the case of our patient. Due to variable sensitivity for different commercially available assays, a negative serum or urine antigen test does not exclude the diagnosis of histoplasmosis [12]. 

BCNE is generally defined as IE without positive blood cultures, present in up to 30% of IE cases [13]. This is mainly due to antibiotic exposure before blood cultures are collected or infection with a fastidious or non-culturable pathogen. If after 72 hours (as in the case of our patient), all blood cultures are negative, lab testing expanded to include the latter organisms is recommended [13]

Our patient did not undergo testing for *Tropheryma whipplei*, although a known cause of BCNE, because he had no gastrointestinal symptoms and had a good response to anti-fungal therapy. Non-infectious causes like non-bacterial thrombotic (marantic) endocarditis (NBTE) were deemed unlikely as he was up-to-date on his age-appropriate cancer screening and his overall presentation was not suggestive of autoimmune disease. He did not undergo autoimmune serology as a result. 

Without appropriate treatment, *Histoplasma* endocarditis is uniformly fatal. A systematic literature review by Boyanton et al., encompassing 60 individual cases from 1940 to 2020, revealed 100% mortality for patients who failed to receive both anti-fungal and surgical treatment for both native and prosthetic valve endocarditis [8]. For those who received anti-fungal therapy alone, like our patient, the mortality was 50%. The general recommendation is for a two-phase treatment with anti-fungal therapy and valvular surgery [14]. The reported mortality in a review of 60 cases was 0% for patients who received anti-fungal and surgical treatment [8]. Fungal endocarditis is deemed a stand-alone indication for surgical replacement of the infected valve [14]. 

Parenteral amphotericin B (preferably one of the lipid formulations) or an azole anti-fungal is recommended for pharmacologic treatment. Our patient was treated with liposomal amphotericin B for one week, in keeping with data to support rapid clearance of fungemia [15]. Although a very large (>15 mm) vegetation is an indication for valvular surgery [16], the patient in the current report was considered a poor surgical candidate due to his multiple co-morbidities and hence discharged on itraconazole with close Infectious Disease specialist follow-up. Lifelong oral anti-fungal suppressive therapy is recommended for such patients with good response to induction medical therapy but who are not deemed to be appropriate surgical candidates for valve replacement [14].

The rarity of* Histoplasma *endocarditis suggests that diagnostic guidelines remain largely based on case series and retrospective reviews. Future studies may help refine diagnostic algorithms that integrate advanced serological testing and molecular methods, enhancing early detection and tailoring therapy more precisely for at-risk populations.

## Conclusions

*Histoplasma capsulatum* is a rare but potentially fatal cause of BCNE, particularly in patients with immunosuppression, ESRD, and occupational exposures. This case emphasizes the importance of maintaining a high index of suspicion for fungal pathogens in at-risk populations and adding fungal serologies early in BCNE workup. Despite negative blood cultures and urine antigen, serologic testing was diagnostic, underlining its utility in current fungal diagnostics. Our patient was discharged in a stable condition on long-term itraconazole as surgery was precluded by his comorbidities. Given that the true prevalence of *Histoplasma* endocarditis is likely underestimated, improved clinical awareness, especially in endemic regions, and standardized diagnostic protocols are essential for timely diagnosis and treatment.
